# DISTAL FEMUR HEMIEPIPHYSIODESIS IN KNEE RECURVATUM: A NEW SURGICAL TECHNIQUE

**DOI:** 10.1590/1413-785220233105e268307

**Published:** 2023-12-18

**Authors:** NEI BOTTER MONTENEGRO, TALISSA OLIVEIRA GENEROSO, BÁRBARA LÍVIA CORRÊA SERAFIM, AMÂNCIO RAMALHO

**Affiliations:** 1 Universidade de Sao Paulo, Faculdade de Medicina, Hospital das Clinicas, Instituto de Ortopedia e Traumatologia IOT HCFMUSP, Grupo de Ortopedia Pediatrica, Sao Paulo, SP, Brazil; 2 Hospital Israelita Albert Einstein, Grupo de Ortopedia Pediatrica, Sao Paulo, SP, Brazil

**Keywords:** Bone Retroversion, Orthopedic Procedures, Minimally Invasive Surgical Procedures, Knee Joint, Growth and Development, Growth Plate, Retroversão Óssea, Procedimentos Ortopédicos, Procedimentos Cirúrgicos Minimamente Invasivos, Articulação do Joelho, Crescimento e Desenvolvimento, Lâmina de Crescimento

## Abstract

**Introduction:**

The genu recurvatum is characterized by a hyperextension deformity of the knee in the sagittal plane. Among its causes are conditions such as arthrogryposis, cerebral palsy, poliomyelitis, sequelae of tibial tuberosity fracture and some syndromes with generalized joint hypermobility. Treatment of this deformity can be challenging and, to date, aggressive methods such as femur or tibial osteotomies are the most used for its correction.

**Objective:**

This study aimed to describe a new surgical technique for correcting genu recurvatum.

**Methods:**

This is a prospective clinical study of children who underwent posterior hemiepiphysiodesis of the distal femur with transphyseal screws.

**Results:**

The approach proved to be safe and useful for genu recurvatum deformities, with femoral or articular apex.

**Conclusion:**

This approach shows great potential for correcting genu recurvatum in the developing skeleton, being an excellent alternative to the more aggressive methods currently used to treat this deformity. ***Level of evidence IV, Case Series.***

## RESUMO

**Introdução:**

O joelho recurvato é caracterizado por uma deformidade em hiperextensão do joelho no plano sagital. Entre suas causas, estão condições como artrogripose, paralisia cerebral, poliomielite, sequelas de fratura da tuberosidade da tíbia e algumas síndromes com hipermobilidade articular generalizada. O tratamento dessa deformidade pode ser desafiador e, até o momento, métodos agressivos como osteotomias do fêmur ou da tíbia são os mais utilizados para sua correção.

**Objetivo:**

Descrever uma nova técnica cirúrgica de correção do genu recurvatum.

**Métodos:**

Estudo clínico prospectivo de crianças submetidas à hemiepifisiodese posterior do fêmur distal com parafusos transfisários.

**Resultados:**

A técnica se mostrou segura e útil para as deformidades em recurvato do joelho, com ápice femoral ou articular.

**Conclusão:**

Essa técnica apresenta grande potencial de correção do joelho recurvato no esqueleto imaturo, sendo uma excelente alternativa aos métodos mais agressivos atualmente utilizados para o tratamento dessa deformidade. ***Nível de Evidência IV, Série de Casos***

## INTRODUCTION

Genu recurvatum, also known as knee recurvatum, is characterized as a hyperextension deformity of the knee in the sagittal plane and, when left untreated, is associated with short- and long-term complications such as joint pain and early gonarthrosis. At the extreme end of the spectrum, there may even be anterior dislocation of the knee.^1^ ^), (^ ^2^ ^), (^ ^3^

Congenital recurvatum, an uncommon deformity of the knee in children, is caused by conditions such as arthrogryposis, ^(^ ^4^ cerebral palsy, ^(^ ^5^ ^)-(^ ^7^ poliomyelitis, sequelae of tibial tuberosity fractures, ^(^ ^8^ ^),(^ ^9^ and some syndromes with generalized joint hypermobility. ^(^ ^1^ ^),(^ ^10^

It is important to note that the treatment of genu recurvatum is challenging. When the deformity is significant and surgical correction is indicated, soft tissue surgical procedures can be used, such as quadricepsplasty^11^ and hamstring lengthening, ^(^ ^6^ ^),(^ ^12^ as well as osteotomies of the distal femur and proximal tibia with internal^13^ or external^14^ ^)-(^ ^16^ fixation to correct bone alignment. The surgical procedures mentioned above are aggressive and require a long recovery time, in addition to risks such as neurovascular injury, compartment syndrome, and infections.

In this context, in search of less aggressive methods with excellent potential for correcting this angular deformity, we used guided growth with posterior hemiepiphysiodesis of the distal femur to correct recurvatum. This article presents a surgical technique using two transphyseal cannulated screws inserted into the posterior portion of the distal femoral physis and the clinical and radiographic results of three patients treated with this method.

## METHODS

### Casuistry

Three patients were treated, totaling four knees with genu recurvatum, three on the left side and one on the right side. Two had arthrogryposis multiplex congenita (one patient with bilateral recurvatum and one with unilateral recurvatum) and one had a unilateral deformity caused by joint hypermobility.

All the patients’ legal guardians signed an informed consent form before the surgical treatment and the procedures followed the norms of the Human Research Ethics Committee with the protocol approved by the Research Ethics Committee of the Hospital das Clínicas of the Faculty of Medicine of the University of São Paulo under number 4.334.540.

### Surgical technique

With the patient in horizontal dorsal decubitus, two 1 cm longitudinal incisions were made on the anterior surface of the distal thigh and blunt dissection was performed through the quadriceps muscle to the anterior surface of the distal femur, in an area proximal to the epiphyseal disc.

Using percutaneous methods and fluoroscopic guidance with images in the coronal and sagittal planes, two guide wires, one for each incision, were passed from anterior to posterior and proximal to distal. They crossed the distal femoral epiphyseal disk in its posterior third, close to the subchondral limit of the medial and lateral femoral condyles.

**Figure 1:**
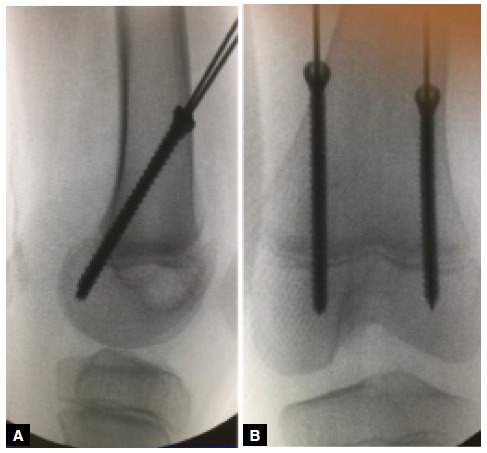
Intraoperative control of the posterior hemiepiphysiodesis of the distal femur, with two cannulated screws guided by metallic wires (A - side view; B - anteroposterior view), for the treatment of genu recurvatum deformity due to joint hypermobility in a 9-year-old patient.

Two cannulated screws with a diameter of 5.5 mm, threaded along their entire length, were inserted through the guide wires, with the tips of the screws positioned completely within the distal femoral epiphysis (Figure 1A and 1B). The subcutaneous tissue and skin were then sutured.

The patients were released from knee mobilization and limb loading immediately after surgery.

Every four months, the degree of deformity was clinically and radiographically assessed until its complete correction, at which point the screws were then removed.

## RESULTS

The surgical treatment was performed and followed clinically and radiographically until the correction of the deformity. The average time to correct the deformity was 15 months, with a minimum follow-up of 1 year. No peri- or post-operative complications or recurrence of the deformity occurred in any of the cases described.

**Figure 2:**
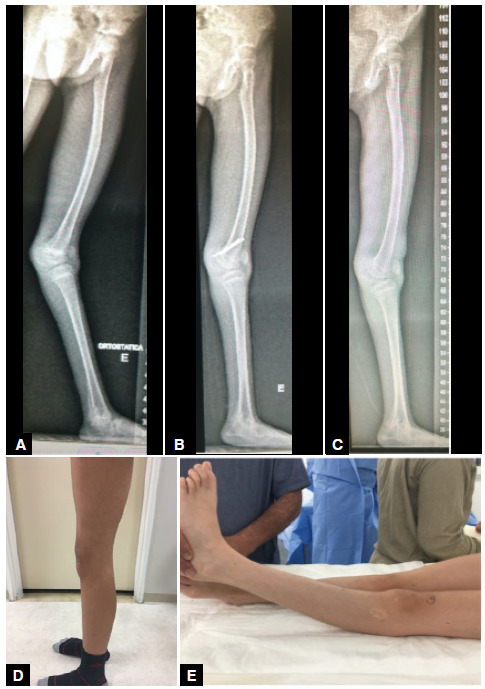
(A) Panoramic radiograph of the left lower limb in profile, a 9-yearold female patient, demonstrating a 32-degree knee recurvatum deformity due to joint hypermobility; (B) Panoramic radiograph of the left lower limb in profile, 1 year and 1 month after surgical treatment with posterior epiphysiodesis of the distal femur, showing correction of the knee recurvatum deformity; (C) Panoramic radiograph in profile, 1 year and 2 months after the removal of the screws from the posterior epiphysiodesis of the distal femur, with maintenance of the correction of the left knee recurvatum deformity; (D) Preoperative photograph of the left lower limb in profile, demonstrating the left knee recurvatum deformity; (E) Photograph taken 1 year after the correction of the left knee recurvatum deformity by posterior epiphysiodesis of the distal femur.

**Figure 3:**
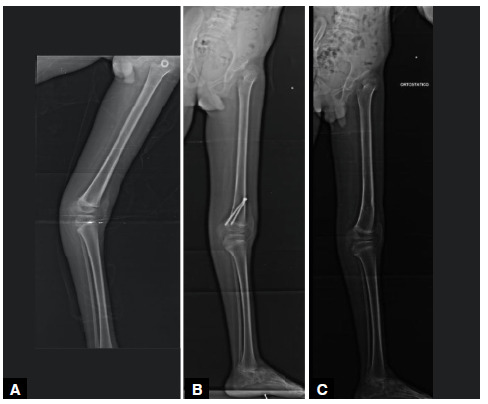
(A) Panoramic radiograph of the left lower limb in profile, a 10-year-old male patient, with a 33-degree knee recurvatum deformity due to arthrogryposis; (B) Panoramic radiograph of the left lower limb in profile, 1 year and 5 months after surgical treatment with posterior epiphysiodesis of the distal femur, showing correction of the deformity; (C) Panoramic radiograph in profile, 1 year and 1 month after the removal of the epiphysiodesis, with maintenance of the correction.

**Figure 4:**
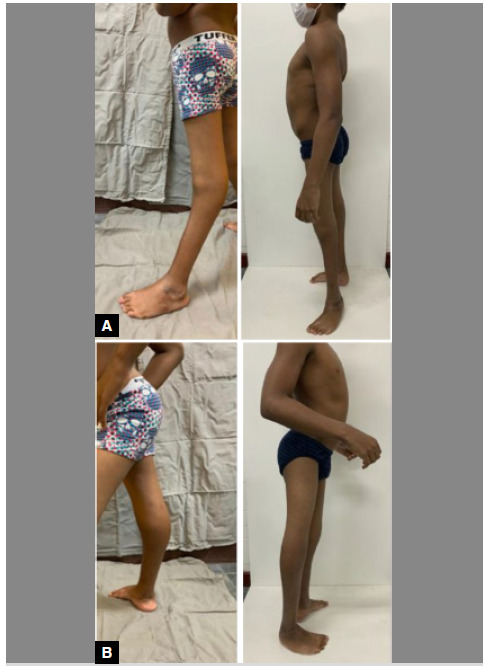
Pre- and post-operative clinical photographs of the left (A) and right (B) lower limbs in profile, showing correction of the recurvatum deformity by posterior epiphysiodesis of the distal femur in a patient with artrogiposys and mild hemiparetic cerebral palsy.

The average correction of the femorotibial angle in the sagittal plane was 26°, with a maximum angle of 32° and a minimum of 18° (Figures 2, 3, and 4).

## DISCUSSION

The knee recurvatum, or hyperextension (genu recurvatum), can be caused by bone deformities affecting the tibia or femur, neuro-orthopaedic diseases, ^(^ ^6^ traumatic anterior tibial fractures or epiphysiodesis, infections, iatrogenies, ^(^ ^9^ capsular-ligament malformations due to arthrogryposis and syndromes with joint hypermobility.

The treatment for this deformity is indicated based on the clinical implications for gait and joint function, although hyperextension has less impact than a fixed knee in flexion.

The clinical presentation is characteristic, with posterior angulation of the knee, either unilateral or bilateral, depending on the underlying cause. The patient may experience claudication while walking, especially when it is unilateral or asymmetrical. The orthostatic radiographic analysis with the knees in maximum extension defines the origin of the deformity (bone, joint or mixed) and allows the calculation of the tibiofemoral deformity angle using a goniometer. The indications for surgical correction depend on this analysis. The non-surgical treatment modalities for knee recurvatum include physical therapy, serial casting, and orthoses. ^(^ ^17^ ^),(^ ^18^ Surgery is reserved for situations in which the deformity is more resistant and also as part of the overall treatment plan, which may include correcting deformities in the foot^19^ and hip. ^(^ ^6^ Among the surgical options available, quadriceps tenotomies, quadricepsplasty, and shortening or opening wedge osteotomies with the addition of bone graft can be considered. In cases of bone deformities, supracondylar osteotomies of the femur with the removal of a posterior wedge, aimed at normalizing the angle between the diaphysis and the intercondylar groove, is a described surgical option. Another treatment option used is anterior opening wedge tibial osteotomy, above the tibial tuberosity, and the insertion of bone graft. ^(^ ^8^ In the latter, it is important to avoid distal displacement of the patella by reinserting the patellar tendon proximally. Osteotomies can be combined with posterior capsular repair, ^(^ ^20^ indicated mainly in cases that present premature closure of the anterior portion of the epiphyseal disc. ^(^ ^9^ ^),(^ ^15^ The most used surgical treatment for knee recurvatum is osteotomies of the distal femur or proximal tibia. ^(^ ^13^ However, these procedures have higher morbidity and risk of complications, requiring a search for less invasive, safer, and more effective methods.

This study reports on the surgical treatment of the knee recurvatum using guided growth with posterior hemiepiphysiodesis of the distal femur using two screws. This method is indicated for deformities caused by capsuloligamentous hyperextension and arthrogryposis. This treatment is not indicated when premature closure of the anterior physeal plate is observed, femoral or tibial, due to any etiology. ^(^ ^15^ Occasionally, in these situations, the posterior epiphysiodesis of the distal femur could only be indicated to reduce the progression of the deformity during the residual growth of the patient.

For the provisional posterior epiphysiodesis of the distal femur surgery, two transphyseal cannulated screws were inserted in the sagittal plane to allow anterior growth of the distal femur. This was based on the reports by Métaizeau et al. ^(^ ^21^ on the guided growth techniques described for correcting deformities in the coronal plane (varus and valgus).

The patients were released for immediate loading. Deformities were monitored by clinical and radiographic evaluations every four months until the recurvatum deformities of the treated knees reached full correction; then, the screws were removed to release the linear growth of the distal femur. It is a minimally invasive, reversible method with a low rate of complications, does not require post-operative immobilization, and most patients are able to walk after the procedure and return to their normal activities. It is important to emphasize the need for follow-up at short intervals, to define the exact moment when the screws should be removed, avoiding overcorrection with inversion of the deformity.

Among the causes of this deformity, which can be treated by the method described in this article, arthrogryposis is a condition that is present from birth and is seen in different diseases, all of which have in common the presence of stiffness and multiple joint deformities. The clinical presentation is diverse and the functional prognosis depends on the etiology, which differentiates the therapeutic options from case to case. In arthrogryposis, knee involvement is very common (38-90% of patients with amyoplasia), ranging from soft tissue contractures (in flexion or hyperextension) to instability, subluxation, or femorotibial dislocation. Flexion contractures are more common and disabling, with significant resistance to treatment and a high recurrence rate. ^(^ ^4^ The prognosis for ambulation is better with recurvatum deformities. According to the literature, nonoperative treatment of knee recurvatum in arthrogryposis with passive mobilization and orthoses fails in about one third of cases. Surgical intervention is recommended, particularly when the knee flexion is limited to 35° or less. According to Lampasi, Antonioli, and Donzelli, ^(^ ^17^ the most used methods to date are quadricepsplasty and femoral shortening and flexion osteotomies, procedures with a higher complication rate than the percutaneous hemiepiphysiodesis using transphyseal screws described in this study, with which we have obtained good results.

Patients with knee recurvatum due to ligament laxity have few options for physical therapy or surgical soft-tissue correction, and osteotomy is reserved for patients with significant gait limitations. The posterior femoral hemiepiphysiodesis presented in this study is undoubtedly a less aggressive surgical alternative with lower risks and a progressive and permanent correction after screw removal.

Guided growth is used as a treatment method for lower limb deformities in the sagittal plane. Jorneau, ^(^ ^22^ Klatt and Stevens, ^(^ ^14^ and Stevens, Stephens, and Rothberg^23^ described correction of the knee in flexion with guided growth by anterior hemiepiphysiodesis of the distal femur with two plates (Eight Plate).

In 2021, Stevens, Stephens, and Rothberg^23^ also described guided growth of the tibial recurvatum by posterior epiphysiodesis of the proximal tibia using the Eight-Plate, with excellent results. Kievit, van Duijvenbode, and Stavenuiter^24^ reported a case of knee recurvatum as a complication of treatment of lower limb length discrepancy with temporary epiphysiodesis of the distal femur and proximal tibia using Eight-Plate. The hypothesis is that the recurvate was caused by a very anterior positioning of the plates, and then the correction of the recurved deformity was obtained with the surgical reapproach and posterior replacement of the plates in the distal femur.

No studies have been found on treatment of genu recurvatum using posterior hemiepiphysiodesis of the distal femur with transphyseal screws, as described in this study.

## CONCLUSION

Posterior hemiepiphysiodesis of the distal femur with transphyseal screws proved to be a safe and very useful approach for recurvatum deformities of the knee whose apex is in the femur or associated with joint hypermobility. This approach shows great potential for correcting the knee recurvatum in the developing skeleton and serves as an excellent alternative to the more aggressive methods currently employed to treat this deformity.

## References

[1] Herring JA (2022). Tachdjian's pediatric orthopaedics: from the Texas Scottish Rite Hospital for Children.

[2] Mehrafshan M, Wicart P, Ramanoudjame M, Seringe R, Glorion C, Rampal V (2016). Congenital dislocation of the knee at birth - Part I Clinical signs and classification. Orthop Traumatol Surg Res.

[3] Ooishi T, Sugioka Y, Matsumoto S, Fujii T (1993). Congenital dislocation of the knee Its pathologic features and treatment. Clin Orthop Relat Res.

[4] Thomas B, Schopler S, Wood W, Oppenheim WL (1985). The knee in arthrogryposis. Clin Orthop Relat Res.

[5] Bauer J, Patrick Do K, Feng J, Pierce R, Aiona M (2019). Knee recurvatum in children with spastic diplegic cerebral palsy. J Pediatr Orthop.

[6] Gugenheim JJ, Rosenthal RK, Simon SR (1979). Knee flexion deformities and genu recurvatum in cerebral palsy roentgenographic findings. Dev Med Child Neurol.

[7] Klotz MCM, Heitzmann DWW, Wolf SI, Niklasch M, Maier MW, Dreher T (2016). The influence of timing of knee recurvatum on surgical outcome in cerebral palsy. Res Dev Disabil.

[8] Blount WP (1954). Fractures in children.

[9] Ishikawa H, Abrahan LM, Hirohata K (1984). Genu recurvatum a complication of prolonged femoral skeletal traction. Arch Orthop Trauma Surg (1978).

[10] Segev E, Hendel D, Wientroub S (2002). Genu recurvatum in an adolescent girl hypothetical etiology and treatment considerations. A case report. J Pediatr Orthop B.

[11] Fiogbe MA, Gbenou AS, Magnidet ER, Biaou O (2013). Distal quadricepsplasty in children 88 cases of retractile fibrosis following intramuscular injections treated in Benin. Orthop Traumatol Surg Res.

[12] Dal Monte A, Manes E, Marchiodi L, Rubbini L (1982). Tenomyoplasty of the flexor muscles in the surgical treatment of congenital recurvatum, subluxation and dislocation of the knee. Ital J Orthop Traumatol.

[13] Bowen JR, Morley DC, McInerny V, MacEwen GD (1983). Treatment of genu recurvatum by proximal tibial closing-wedge/anterior displacement osteotomy. Clin Orthop Relat Res.

[14] Klatt J, Stevens PM (2008). Guided growth for fixed knee flexion deformity. J Pediatr Orthop.

[15] Olerud C, Danckwardt-Lillieström G, Olerud S (1986). Genu recurvatum caused by partial growth arrest of the proximal tibial physis simultaneous correction and lengthening with physeal distraction. A report of two cases. Arch Orthop Trauma Surg (1978).

[16] Manohar Babu KV, Fassier F, Rendon JS, Saran N, Hamdy RC (2012). Correction of proximal tibial recurvatum using the Ilizarov technique. J Pediatr Orthop.

[17] Lampasi M, Antonioli D, Donzelli O (2012). Management of knee deformities in children with arthrogryposis. Musculoskelet Surg.

[18] Nuzzo RM (1986). A simple treatment of genu recurvatum in ataxic and athetoid cerebral palsy. Orthopedics.

[19] Svehlík M, Zwick EB, Steinwender G, Saraph V, Linhart WE (2010). Genu recurvatum in cerebral palsy--part A influence of dynamic and fixed equinus deformity on the timing of knee recurvatum in children with cerebral palsy. J Pediatr Orthop B.

[20] Perry J, O'Brien JP, Hodgson AR (1976). Triple tenodesis of the knee A soft-tissue operation for the correction of paralytic genu recurvatum. J Bone Joint Surg Am.

[21] Métaizeau JP, Wong-Chung JM, Bertrand H, Pasquier P (1998). Percutaneous epiphysiodesis using transphyseal screws (PETS). J Pediatr Orthop.

[22] Journeau P (2020). Update on guided growth concepts around the knee in children. Orthop Traumatol Surg Res.

[23] Stevens P, Stephens A, Rothberg D (2021). Guided growth for tibial recurvatum. Strategies Trauma Limb Reconstr.

[24] Kievit AJ, van Duijvenbode DC, Stavenuiter MHJ (2013). The successful treatment of genu recurvatum as a complication following eight-Plate epiphysiodesis in a 10-year-old girl a case report with a 3.5-year follow-up. J Pediatr Orthop B.

